# Socioeconomic status and survival after stroke – using mediation and sensitivity analyses to assess the effect of stroke severity and unmeasured confounding

**DOI:** 10.1186/s12889-020-08629-1

**Published:** 2020-04-25

**Authors:** Anita Lindmark, Bo Norrving, Marie Eriksson

**Affiliations:** 1 Department of Statistics, Umeå School of Business, Economics and Statistics, Umeå University, Umeå, Sweden; 2 Department of Neurology, Lund University, Lund, Sweden; 3 Department of Statistics, Umeå School of Business, Economics and Statistics, Umeå University, Umeå, Sweden

**Keywords:** Stroke, Income, Socioeconomic factors, Mediation, Direct effect, Indirect effect, Sensitivity analysis, Unmeasured confounding

## Abstract

**Background:**

Although it has been established that low socioeconomic status is linked to increased risk of death after stroke, the mechanisms behind this link are still unclear. In this study we aim to shed light on the relationship between income level and survival after stroke by investigating the extent to which differences in stroke severity account for differences in survival.

**Methods:**

The study was based on patients registered in Riksstroke (the Swedish stroke register) with first time ischemic stroke (*n* = 51,159) or intracerebral hemorrhage (*n* = 6777) in 2009–2012. We used causal mediation analysis to decompose the effect of low income on 3-month case fatality into a direct effect and an indirect effect due to stroke severity. Since causal mediation analysis relies on strong assumptions regarding residual confounding of the relationships involved, recently developed methods for sensitivity analysis were used to assess the robustness of the results to unobserved confounding.

**Results:**

After adjustment for observed confounders, patients in the lowest income tertile had a 3.2% (95% CI: 0.9–5.4%) increased absolute risk of 3-month case fatality after intracerebral hemorrhage compared to patients in the two highest tertiles. The corresponding increase for case fatality after ischemic stroke was 1% (0.4–1.5%). The indirect effect of low income, mediated by stroke severity, was 1.8% (0.7–2.9%) for intracerebral hemorrhage and 0.4% (0.2–0.6%) for ischemic stroke. Unobserved confounders affecting the risk of low income, more severe stroke and case fatality in the same directions could explain the indirect effect, but additional adjustment to observed confounders did not alter the conclusions.

**Conclusions:**

This study provides evidence that as much as half of income-related inequalities in stroke case fatality is mediated through differences in stroke severity. Targeting stroke severity could therefore lead to a substantial reduction in inequalities and should be prioritized. Sensitivity analysis suggests that additional adjustment for a confounder of greater impact than age would be required to considerably alter our conclusions.

## Background

There is a well-documented social gradient in health, where socially underprivileged groups have an increased risk of disease and adverse health outcomes [1–3]. This is also true for stroke, the second most common cause of death worldwide according to the World Health Organization (WHO) and a leading cause of disability among adults. Socioeconomic inequalities in stroke incidence, quality of and access to care, and outcome after stroke have been well established globally where low- and middle-income countries, as well as socially disadvantaged groups within high-income countries have been found to be disproportionately affected by stroke [4].

Patients with low socioeconomic status (SES) are at an increased risk of death after stroke, even in countries with universal access to healthcare [5–10]. The causes behind these differences have not been established but are likely multifaceted, with aspects related both to patient characteristics (risk factors, comorbidities, etc.) and structural problems within the health care system. Patients with low SES have been found to have more severe strokes and higher incidence of cardiovascular risk factors than high SES patients [4, 6, 11]. Examining how much of the effect of SES on survival that is attributable to differences in disease severity may shed light on the extent to which differences are related to pre-stroke (e.g. comorbidities, lifestyle, primary prevention) vs. post stroke factors such as structural differences in acute stroke management and secondary prevention.

By using causal mediation analysis we can separate the effect of SES on post-stroke survival into a direct effect and an indirect effect operating through stroke severity to gain insight into the relative importance of each pathway [12, 13]. The estimation of these effects from observational data requires strong assumptions regarding residual confounding of the relations involved. These assumptions are often difficult or impossible to verify from the observed data and it is therefore crucial to perform sensitivity analyses to assess the robustness of the results to violations [14–18]. The mediation analysis literature has focused almost exclusively on methods to gauge sensitivity to unobserved confounding of the relation between the intermediate variable (mediator) and the outcome, assuming that randomization of the exposure or adjustment for baseline confounders adequately addresses confounding of the exposure-mediator and exposure-outcome relations [14–16, 18, 19]. However, in observational studies no such randomization takes place and it is difficult to guarantee that a sufficiently rich set of baseline confounders has been adjusted for.

The aim of this study is to shed light on the relationship between socioeconomy and survival after stroke by investigating to which extent it can be explained by differences in stroke severity. We focused on short term survival (up to 3 months) after stroke and used individual patient data from Riksstroke, the national Swedish stroke register, in combination with causal mediation analysis. Recent techniques for sensitivity analysis were used to assess the robustness of the results to different types of residual confounding [17].

## Methods

### Data and variable definitions

**Fig. 1:**
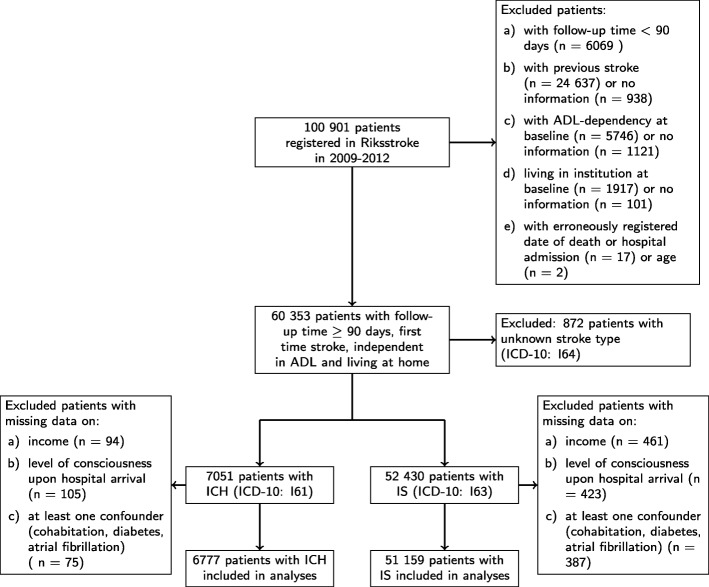
Flow-chart for inclusion of patients in the study

Patients included in the study were registered in Riksstroke with intracerebral hemorrhage, ICH (ICD-10 code I61) or ischemic stroke, IS (ICD-10 code I63) between January 1, 2009 and October 1, 2012. Patients with previous stroke, living in institution, or dependent in ADL (activities of daily living, i.e. unable to manage clothing, toileting or walking unassisted) were excluded from the study. A total of 6777 patients with ICH and 51,159 patients with IS were included in the analyses (Fig. 1). We performed separate analyses for the two main types of stroke, IS and ICH, as these patient groups tend to differ on background variables and present with different severity levels and short term mortality rates [20, 21].

The purpose of Riksstroke is to monitor and support improvement of quality of stroke care in Sweden [22]. Patient-level information is collected during the acute phase at all Swedish hospitals that admit acute stroke patients (currently 72 hospitals) as well as at follow-up 3 months and 1 year after stroke. For this study Riksstroke data were linked with data from the Swedish Cause of Death Register (managed by the Swedish National Board of Health and Welfare) and the LISA database (managed by Statistics Sweden) using the personal identity numbers (Swedish national identification numbers) of the patients. Information on survival was retrieved from the Swedish Cause of Death Register and information on socioeconomic status was retrieved from LISA.

The outcome was death 0–3 months after stroke (defined as death within 90 days after stroke onset).

The choice of proxy for low SES was motivated by previous studies. In a study on a similar cohort we found income to be more closely associated with post-stroke survival than education [5]. This has also been found in register based studies in Denmark and Norway [6, 9]. Based on this we chose to use low income as a proxy for low SES. As in our previous study, we define low income as being in the lowest tertile of the individual’s part of the family disposable income the year before the stroke.

The mediator of interest, stroke severity, was measured using level of consciousness on admission to hospital, based on the Re-action Level Scale (RLS) [23]. An RLS of 1 corresponded to fully conscious and an RLS 2–8 to lowered consciousness.

The covariates included for confounding adjustment were selected based on medical knowledge and availability in Riksstroke. In addition, the definition of a confounder in this scenario is a variable that affects at least two of the exposure, mediator and outcome. Based on these criteria our analyses were adjusted for a set of baseline confounders consisting of patient age (years), sex (male/female), whether or not the patient was living alone at the time of stroke, and the cardiovascular risk factors atrial fibrillation, diabetes and smoking history (smoker/non-smoker/unknown).

We opted not to adjust our main analyses for other SES variables (e.g. education level) as we were interested in a general effect of SES, not specifically a pure income effect. Supplementary analyses with additional adjustment for highest attained education level were performed.

This study was approved by the Ethical Review Board in Umeå.

### Statistical methods

The distributions of characteristics among low and mid/high income patients within each stroke type were compared using Pearson χ^2^ tests (categorical variables) and independent samples t tests (continuous variables).

**Fig. 2:**
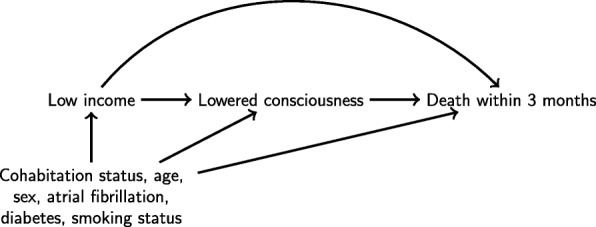
Directed acyclic graph showing the causal relations assumed between the variables in the study

The directed acyclic graph (DAG) in Fig. 2 illustrates the causal relations assumed between the variables in the study. Our exposure of interest was low income, which we assumed had a causal effect on lowered consciousness upon hospital arrival and death 0–3 months after stroke for both stroke types. The baseline confounders were age, sex, living alone, atrial fibrillation, diabetes and smoking history. We decomposed the total effect of low income on death 0–3 months into two parts, an indirect effect of low income on death 0–3 months that acts through lowered consciousness upon arrival and a direct effect that does not act through lowered consciousness. Here we focus on a decomposition of the total effect into the natural indirect effect and the natural direct effect [12, 13]. In our study the indirect effect contrasts the risk of death for patients with low income with their observed level of consciousness to a counterfactual risk where level of consciousness is set to levels that would have been observed had the low income patient been mid/high income. The direct effect contrasts the risk of death for patients with low income and patients with mid/high income if, for all patients, level of consciousness were set to levels that would have been observed had the patient been mid/high income. A more formal definition of these effects is given in Section 1 of Additional file 1.

To estimate the direct and indirect effects we used a regression model based approach [17, 24]. For each stroke type two regression models were fitted, one for the mediator (lowered consciousness upon arrival) given the exposure (low income) and the observed confounders, and one for the outcome (death 0–3 months) given exposure, mediator and observed confounders. As both the mediator and outcome are binary we fit probit regression models. The estimated regression parameters from these models were then used to estimate the direct and indirect effects (see Additional file 1, Section 1 for details).

The estimates of the direct and indirect effects are valid under the assumption that there is no residual confounding of the exposure-mediator, mediator-outcome or exposure-outcome relations and provided that the regression models are correctly specified [24]. See Fig. 1 in Additional file 1 for a DAG illustrating the three types of residual confounding. We also assume that there is no confounder of the mediator-outcome relation (observed or unobserved) that is caused by the exposure.

To assess the robustness of our results to the presence of residual confounding we performed sensitivity analyses using a method introduced in Lindmark et al. [17] We use a *sensitivity parameter* to quantify the effect of a possible unobserved confounder. The goal is then to capture the effect that would have been obtained had we been able to adjust for the unobserved confounder.

For our sensitivity parameter we use the fact that omitting a confounder from our regression models means that the error terms in these models will depend on the omitted confounder and therefore be correlated. Negative correlations correspond to an unobserved confounder that affects both variables of interest in opposite directions (e.g. increasing the risk of the mediator but decreasing the risk of the outcome). Positive correlations correspond to the unobserved confounder affecting both variables of interest in the same direction, either increasing the risk of both or decreasing the risk of both. The larger the absolute value of the correlation (closer to − 1 or + 1), the stronger the effect of the unobserved confounder, with a correlation of 0 indicating no unobserved confounding.

We performed three separate sensitivity analyses, one for each type of confounding. For mediator-outcome confounding the sensitivity parameter is the correlation between the error terms of the mediator and outcome models. For unobserved exposure-mediator and exposure-outcome confounding the sensitivity parameters are the correlations between a model for the exposure given the observed confounders and the mediator and outcome models, respectively.

In the sensitivity analysis we obtained estimates of the direct and indirect effects under a given level of unobserved confounding (a given value of the sensitivity parameter, for details see Section 2 of Additional file 1). Sensitivity analyses are sometimes criticized for being subject to the investigators choice of sensitivity parameter values. To increase objectivity we report the results of the sensitivity analyses based on recommendations in VanderWeele [25]:

i. Estimates are reported across a wide range of the sensitivity parameter (correlations ranging from − 0.9 to 0.9).
ii. The value of the sensitivity parameter required to reduce the estimated effect to 0 is reported.
iii. The most important observed confounder is identified and the results are compared to the impact of omitting this confounder from the estimation.

All analyses were performed using R (version 3.6) [26]. Both the estimation and the sensitivity analyses were performed using the sensmediation package [27]. See Additional file 2 for R code for the mediation and sensitivity analyses.

## Results

Descriptive statistics by stroke type and income level are displayed in Table 1. Patients with ICH had higher short term case fatality and more severe stroke than patients with IS, but were on average younger and had a lower proportion of risk factors.

**Table 1:** Descriptive statistics by stroke type. Number (%)

	ICH			P^a^	IS			P^a^
	All	Low	Mid/High		All	Low	Mid/High	
N	6777	2304	4473		51,159	17,020	34,139	
		(34.0)	(66.0)			(33.3)	(66.7)	
Death 0–3 months	1888	719	1169	< 0.001	5381	2127	3254	< 0.001
	(27.9)	(31.2)	(26.1)		(10.5)	(12.5)	(9.5)	
Lowered consciousness	2455	930	1525	< 0.001	5544	2153	3391	< 0.001
	(36.2)	(40.4)	(34.1)		(10.8)	(12.6)	(9.9)	
Men	3785	894	2891	< 0.001	26,819	5854	20,965	< 0.001
	(55.9)	(38.9)	(64.6)		(52.4)	(34.4)	(61.4)	
Age, mean	70.9	72.2	70.3	< 0.001	74.2	76.0	73.3	< 0.001
(st. dev.)	(13.8)	(14.5)	(13.3)		(12.4)	(12.5)	(12.2)	
Atrial fibrillation	1218	401	817	0.401	13,325	4748	8577	< 0.001
	(18.0)	(17.4)	(18.3)		(26.0)	(27.9)	(25.1)	
Diabetes	929	321	608	0.728	9661	3310	6351	0.022
	(13.7)	(13.9)	(13.6)		(18.9)	(19.4)	(18.6)	
Smoking status				0.551				< 0.001
Smoker	720	234	486		7760	2450	5310	
	(10.6)	(10.2)	(10.9)		(15.2)	(14.4)	(15.6)	
Unknown	785	276	509		3214	1154	2060	
	(11.6)	(12.0)	(11.4)		(6.3)	(6.8)	(6.0)	
Living alone	2852	1022	1830	0.007	23,453	8327	15,126	< 0.001
	(42.1)	(44.4)	(40.9)		(45.8)	(48.9)	(44.3)	

^a^*P*-values from Pearson’s χ^2^ test (categorical variables) and independent samples t tests (age) comparing low and mid/high income patients

Comparing low income and mid/high income patients within each stroke type we see that the proportion of patients who died 0–3 months and the proportion of patients with lowered level of consciousness was higher among low income patients for both ICH and IS (Table 1). The low income patient group was also to a larger extent female, living alone and on average older than the mid/high income group for both IS and ICH. For IS the low income group also exhibited higher proportions of atrial fibrillation and diabetes compared to the mid/high income group, while the proportion of smokers was lower among low income patients.

The estimated probit regression models for the mediator lowered level of consciousness and the outcome death 0–3 months used for estimation of the indirect and direct effects are shown in Section 1 of Additional file 3. Following recommendations by VanderWeele [25] we initially included an interaction term between income and level of consciousness in the outcome models. As these interaction terms were small in magnitude and the estimated direct and indirect effects were virtually identical with and without interactions we did not include them in further analyses.

The estimated total effect of low income on death within 3 months after stroke was larger for ICH than for IS. For ICH, low income patients had a 3.2% (95% CI: 0.9–5.4%) increased absolute risk of death 0–3 months compared to mid/high income patients even after adjustment for observed confounders. The corresponding increase for IS was 1% (0.4–1.5%).

In the decomposition of the total effect for ICH, the indirect effect of low income, mediated by lowered level of consciousness, was 1.8% (0.7–2.9%) and the direct effect 1.4% (− 0.6–3.3%). For IS the indirect effect was 0.4% (0.2–0.6%) and the direct effect 0.6% (0.1–1.1%). Looking at effect sizes, a large part of the total effect of low income on death 0–3 months after stroke was an indirect effect working through lowered level of consciousness. For ICH over half the effect (57.0%) of income on death within 3 months was explained by lowered consciousness upon arrival. This proportion was smaller for IS but still close to two fifths (38.5%) of the effect of low income was mediated by lowered level of consciousness.

### Effect of possible unmeasured confounding

**Fig. 3:**
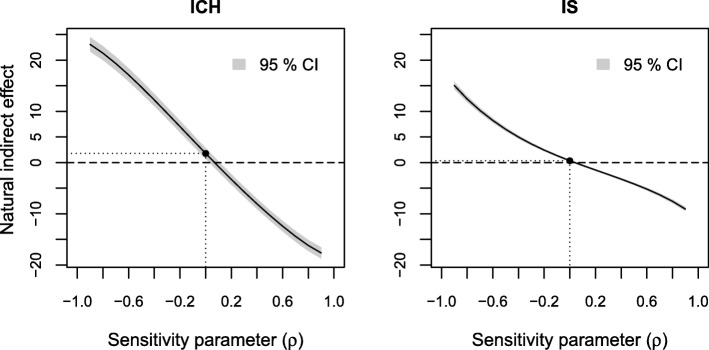
Results of sensitivity analyses to residual confounding of the income-stroke severity relation on the estimated natural indirect effect for ICH and IS

**Fig. 4:**
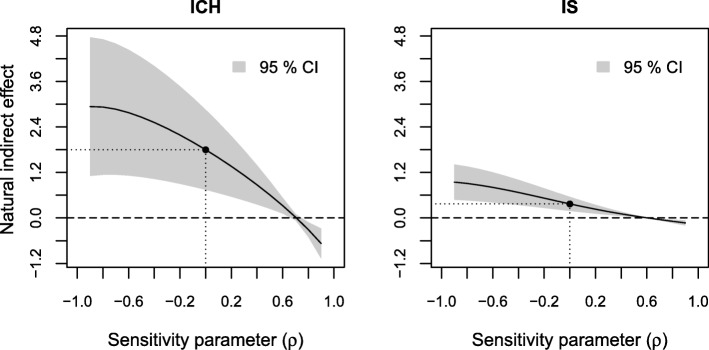
Results of sensitivity analyses to residual confounding of the stroke severity-death 0–3 months relation on the estimated natural indirect effect for ICH and IS

**Fig. 5:**
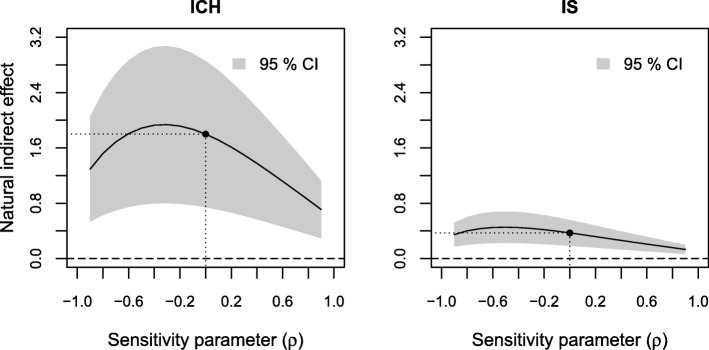
Results of sensitivity analyses to residual confounding of the income-death 0–3 months relation on the estimated natural indirect effect for ICH and IS

The results of sensitivity analyses of the estimated indirect effect to residual confounding are displayed in Figs. 3, 4 and 5, respectively. The sensitivity parameter (denoted ρ) ranged from − 0.9 to 0.9. To assess the sensitivity to residual confounding involving the exposure, an additional probit regression model for low income given the observed confounders was estimated (see Additional file 3, Section 1).

In Fig. 3 we see similar overall patterns across the range of the sensitivity parameter for both ICH and IS. Adjusting for a confounder which affects the risk of low income and death 0–3 months in opposite directions (negative ρ) would increase the estimated indirect effect, while adjusting for a confounder which affects the risk of low income and death 0–3 months in the same direction (positive ρ) would decrease the estimated indirect effect. Moderate positive values of ρ (between 0 and 0.1) would yield a zero effect.

We see a similar but weaker pattern in the sensitivity analysis to residual confounding between the level of consciousness-death 0–3 months relation (Fig. 4). Here, an unobserved confounder would need to have a strong impact (ρ around 0.7 for ICH and ρ around 0.6 for IS) in order for additional adjustment to reduce the estimated indirect effect to 0.

In Fig. 5 we see that the estimated indirect effect does not appear to be sensitive to residual confounding of the income-death 0–3 months relation as it remained relatively constant over the range of the sensitivity parameters for both stroke types.

**Fig. 6:**
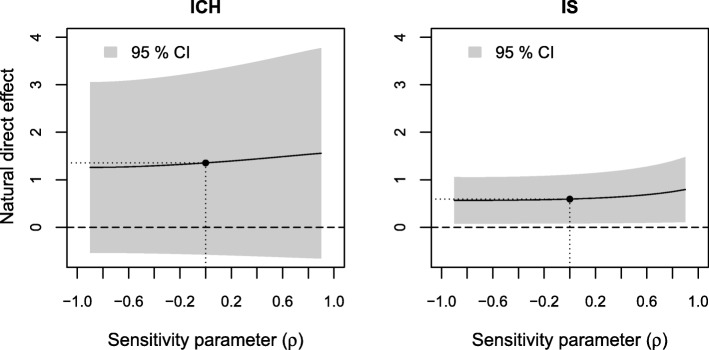
Results of sensitivity analyses to residual confounding of the income-stroke severity relation on the estimated natural direct effect for ICH and IS

**Fig. 7:**
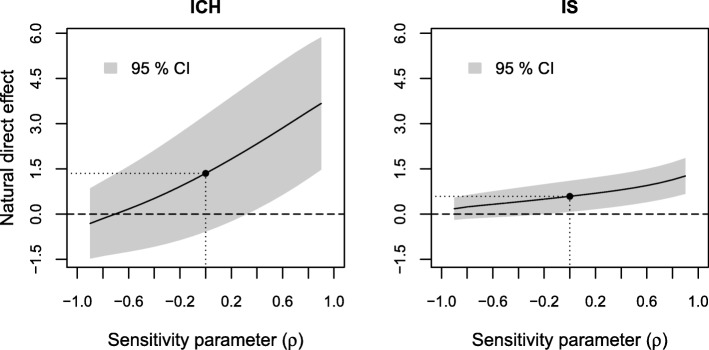
Results of sensitivity analyses to residual confounding of the stroke severity-death 0–3 months relation on the estimated natural direct effect for ICH and IS

**Fig. 8:**
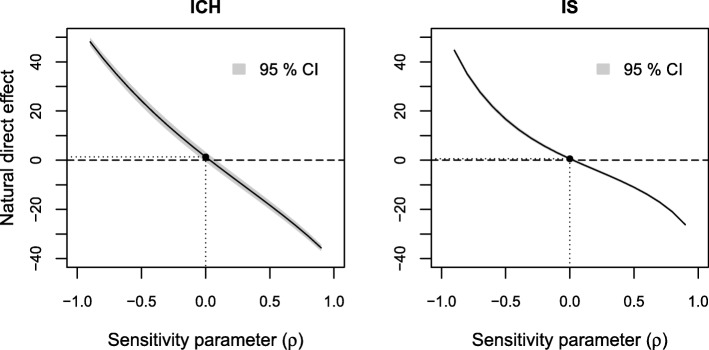
Results of sensitivity analyses to residual confounding of the income-death 0–3 months relation on the estimated natural direct effect for ICH and IS

In the sensitivity analyses for the estimated direct effect we see that the direct effect is not sensitive to residual confounding of the income-level of consciousness relation (Fig. 6). The estimated direct effect increased over the range of the sensitivity parameter in the sensitivity analysis to residual confounding of the level of consciousness-death 0–3 month relation (Fig. 7) but decreased over the range of the sensitivity parameter in the sensitivity analysis to residual confounding of the income-death 0–3 month relation (Fig. 8). For the latter, moderate positive values of ρ (close to 0) would yield a zero effect.

Finally, we looked at the effect of omitting age, the most important observed predictor of income, level of consciousness and death 0–3 months, from our models. When not adjusting for age the estimated indirect effect was 2.0% (95% CI: 0.9–3.1%) and the direct effect 2.1% (0.1–4.1%) for ICH. For IS the indirect effect was 0.6% (0.4–0.8%) and the direct effect was 1.4% (0.9–1.9%) without adjustment for age. That is, additional adjustment for age yielded a modest decrease of the estimated indirect effect and a more substantial decrease of the estimated direct effect and therefore an increase in the proportion mediated for both stroke types. These values cannot be directly translated to correlations in Figs. 3, 4, 5, 6, 7 and 8, since correlation values correspond to looking at each type of confounding in isolation, assuming that the other two types are not present. This assumption does not hold for age, which affects all three relations, but we still get an indication that it would take an unobserved confounder of greater impact than age to considerably alter our conclusions.

## Discussion

Our results showed an increased risk of death within 3 months after stroke for low vs. mid/high income patients with first-time stroke and who were independent in ADL and living at home at the time of stroke. The gradient was especially pronounced for patients with ICH, corresponding to 89 additional deaths per 10,000 patients among low income patients compared to mid/high income patients. For IS the corresponding number was 10 per 10,000 patients. We found that for ICH nearly three fifths and for IS nearly two fifths of the effect operated through stroke severity (lowered consciousness upon hospital arrival).

An effect of low income/SES on death after stroke has been shown in studies from Denmark, England, Canada, Norway, Wales and the USA [6–11, 28, 29]. Several studies on the SES-stroke survival relation have adjusted the analyses for stroke severity, either as confounding adjustment [5–7, 11] or specifically focusing on the attenuation of the estimated SES-survival effect [8]. Earlier studies have also shown that patients with low SES tend to have more severe strokes than high SES patients [4, 6, 11]. However, ours is the first study to estimate the proportional contribution of stroke severity of the effect of SES on survival; we found that the contribution of stroke severity was substantial even after adjustment for other established prognostic factors such as age and atrial fibrillation. Further studies are needed to determine how SES is pathophysiologically linked to stroke severity. Potential factors may e.g. be differences in the early recognition of stroke, time to hospital, and other pre-hospital factors. Further studies should also include further subtyping of ischemic and hemorrhagic stroke, and results from neuroimaging and other diagnostic procedures.

Our results also indicate that a not unsubstantial part of the effect of income on death within 3 months is not mediated by stroke severity. The remaining association could be due to other pathways that link income with survival, e.g. differences in acute stroke management and/or secondary prevention.

In all observational studies there is a risk of residual confounding. Here, we used a recent method for sensitivity analyses [17] to assess the effect of violations of the crucial assumptions about residual confounding of not only the relation between the mediator (lowered consciousness) and the outcome (death within 3 months) but also the relations involving the exposure (low income).

We found that the indirect effect was most sensitive to unobserved confounders that increase both the risk of having low income and having lowered consciousness upon arrival to hospital. The direct effect was most sensitive to unobserved confounders that increase both the risk of having low income and death within 3 months after stroke. Individual linkage of several national registers can provide information on a rich set of possible confounders. Still, genetic factors, life style (e.g. diet, exercise habits, alcohol consumption), and demography (e.g. distance from the patient’s residence to the hospital) may contribute to residual confounding in studies such as ours. Additional adjustment for such confounders could potentially explain the observed effects. We did observe that additional adjustment for the most important observed confounder, age, attenuated the estimated direct effect more than the indirect effect leading to an increased proportion of the total effect mediated by stroke severity. This was also observed with additional adjustment for education level (see below).

In order to maintain a high coverage and participation rate in Riksstroke, the amount of information collected in the register has been kept at a minimum, and does not include detailed information on e.g. comorbidity, stroke location or size. In addition to the potential risk of residual confounding, there are also other limitations to the study. SES is a multidimensional concept that can be measured in different ways. Several previous studies, including one on a similar cohort, found income to be more strongly associated with post-stroke survival than education [5, 6, 9]. As we were interested in a general effect of SES, not specifically a pure effect of low income we opted not to adjust for other SES variables, such as education level, in our analyses. Supplementary analyses with adjustment for education showed an attenuation of the total effect but an increased proportion mediated (see Additional file 3, Section 2). We measure income the year before stroke, focusing on adulthood SES, but there is evidence in favor of a link between childhood SES and adulthood stroke [30] as well as a cumulative effect of SES over the life course [31] that is probably not reflected in our measurement.

We used level of consciousness, based on RLS, upon arrival to hospital as a proxy for stroke severity, our mediator of interest. This measurement was dichotomized into fully conscious and lowered level of consciousness. It is possible that the use of a more comprehensive measurement of stroke severity, such as NIHSS, would give different results. However, level of consciousness has been proven a good proxy for the full NIHSS in predicting death after stroke [32].

Even if it is evident that stroke severity plays an important role in the mechanism explaining the increased risk of death after stroke in low SES groups, the exact proportion mediated may not be generalized to countries with e.g. different demography, and health care settings.

We made assumptions about the directions of associations between the variables in the study. This cannot be directly ascertained from cross sectional data, and was based on clinical reasoning and prior knowledge. A statistical model will always be a simplification of the real world. Here we used mediation analysis to add information on the complex relation between SES and survival. Still, we did not consider e.g. intermediate confounders (confounders of the stroke severity- death within 3 months relation that are affected by income), or several mediating factors. This would require the use of alternative effect definitions and methods and may be a subject for future studies [33–36].

## Conclusions

This study suggests that as much as half of the effect of low SES on increased risk of death operates through stroke severity. Hence, targeting stroke severity could potentially lead to a large reduction in inequalities and should therefore be prioritized.

## Supplementary information

**Additional file 1.** Formal definitions and technical details.

**Additional file 2.** R code.

**Additional file 3.** Tables.

## Data Availability

The data set comprises linked information from Riksstroke, Statistics Sweden and the National Board of Health and Welfare and cannot be made publically available due to Swedish legislation. Requests to access the dataset may be sent to Riksstroke, Statistics Sweden and the National Board of Health and Welfare after obtaining the appropriate ethics approval. Code for the main analyses are available in Additional file 2 and any additional code related to the results of the study are available from the corresponding author upon reasonable request.

## Abbreviations

WHO: World Health Organization
SES: Socioeconomic status
ICH: Intracerebral hemorrhage
IS: Ischemic stroke
ADL: Activities of daily living
LISA: Longitudinal Integration Database for Health Insurance and Labor Market Studies
RLS: Reaction Level Scale
DAG: Directed acyclic graph
st. dev: Standard deviation
CI: Confidence interval
USA: United States of America
NIHSS: National Institutes of Health Stroke scale
