# Supplementary material for: Socioeconomic status and survival after stroke – using mediation and sensitivity analyses to assess the effect of stroke severity and unmeasured confounding
Source: BMC Public Health. 2020 Apr 25;20:554. doi: 10.1186/s12889-020-08629-1 (PMC7183587; doi:10.1186/s12889-020-08629-1)
Supplement: Supplementary file 1 — Additional file 1. Formal definitions and technical details. [file 12889_2020_8629_MOESM1_ESM.pdf]

Additional file 1: Formal definitions and technical details

Article title: Socioeconomic status and survival after stroke - using mediation and sensitivity analyses to assess the effect of stroke severity and unmeasured confounding
Authors: Anita Lindmark, Bo Norrving, Marie Eriksson

1 Definition and estimation of natural direct and indirect effects

Definitions of direct and indirect effects within the causal inference framework have been given by Robins and Greenland [1] and Pearl [2]. Here we present the definitions of the effects used in the study. Let Z_i, M_i, Y_i denote the exposure, mediator and outcome for patient i.

First we outline the potential outcomes used to define the effects. Let Z be a binary exposure where $Z = 1$ if exposed and $Z = 0$ if unexposed. Let $Y_i(z)$ denote the potential outcome for patient i, i.e. the outcome that would have been observed for patient i if that patient had been exposed at level z, $z = 0, 1$. Similarly we define the potential mediator for patient i under exposure level z as $M_i(z)$. Finally, we define the potential outcome for patient i had that patient experienced exposure level z and mediator level m as $Y_i(z, m)$ and the *composite potential outcome* if the exposure were set to the value z and the mediator were set to its value under exposure level z' as $Y_i(z, M_i(z'))$, $z, z' = 0, 1$.

We define the *natural direct effect* on the mean difference scale as

$$\text{NDE} = E\left[Y_i(1, M_i(0)) - Y_i(0, M_i(0))\right],$$

the effect of Z on Y if the mediator were allowed to vary as it would naturally if all patients in the population were unexposed.

The *natural indirect effect* on the mean difference scale is defined as

$$\text{NIE} = E\left[Y_i(1, M_i(1)) - Y_i(1, M_i(0))\right],$$

the effect on Y when, keeping the exposure fixed at $Z_i = 1$ in the whole population, allowing the mediator to change from its potential value when $Z_i = 0$ to its potential value when $Z_i = 1$.

The *total effect* of the exposure on the outcome, $\text{TE} = E\left[Y_i(1) - Y_i(0)\right]$ can be decomposed as $\text{TE} = \text{NDE} + \text{NIE}$.

To estimate the natural direct and indirect effects from observed data we need to assume that there is no unobserved exposure-mediator, mediator-outcome or exposure-outcome confounding, and that there is no mediator-outcome confounder that is affected by the exposure (this has been formulated by e.g. Imai et al [3] who refer to it as *sequential ignorability*). We also assume *positivity*, *consistency* and *no interference*, described elsewhere [4, 5].

We use an estimation approach based on parametric regression models. Let Z_i, M_i and Y_i be the observed exposure, mediator and outcome for patient i, all binary. Let $\boldsymbol{X}_i$ be the vector of baseline confounders for patient i. Also, let $I(A > 0)$ be an indicator variable that takes the value 1 if $A > 0$ and 0 otherwise. We then assume that $M_i = I(M_i^* > 0)$, where M_i^* is a latent variable modeled as

$$M_i^* = \beta_0 + \beta_1 Z_i + \boldsymbol{\beta}_2^\top \boldsymbol{X}_i + \eta_i. \tag{1}$$

We also assume that $Y_i = I(Y_i^* > 0)$, where Y_i^* is a latent variable modeled as

$$Y_i^* = \theta_0 + \theta_1 Z_i + \theta_2 M_i + \theta_3 Z_i M_i + \boldsymbol{\theta}_4^\top \boldsymbol{X}_i + \xi_i. \tag{2}$$

The error terms η_i and ξ_i are assumed to be i.i.d. standard normal random variables, giving probit mediator and outcome models.

Assuming that these models are correctly specified, the natural direct and indirect effects are given by the expressions [6]

$$\begin{aligned}\text{NDE} = {} & \frac{1}{n}\sum_{i=1}^{n}\left\{\Phi\left(\theta_0 + \theta_1 + \boldsymbol{\theta}_4'\boldsymbol{x}_i\right) - \Phi\left(\theta_0 + \boldsymbol{\theta}_4'\boldsymbol{x}_i\right)\right\}\left(1 - \Phi\left(\beta_0 + \boldsymbol{\beta}_2'\boldsymbol{x}_i\right)\right) + \\ & \frac{1}{n}\sum_{i=1}^{n}\left\{\Phi\left(\theta_0 + \theta_1 + \theta_2 + \theta_3 + \boldsymbol{\theta}_4'\boldsymbol{x}_i\right) - \Phi\left(\theta_0 + \theta_2 + \boldsymbol{\theta}_4'\boldsymbol{x}_i\right)\right\} \times \Phi\left(\beta_0 + \boldsymbol{\beta}_2'\boldsymbol{x}_i\right),\end{aligned} \tag{3}$$

$$\begin{aligned}\text{NIE} = {} & \frac{1}{n}\sum_{i=1}^{n}\left\{\Phi\left(\theta_0 + \theta_1 + \theta_2 + \theta_3 + \boldsymbol{\theta}_4'\boldsymbol{x}_i\right) - \Phi\left(\theta_0 + \theta_1 + \boldsymbol{\theta}_4'\boldsymbol{x}_i\right)\right\} \times \\ & \left\{\Phi\left(\beta_0 + \beta_1 + \boldsymbol{\beta}_2'\boldsymbol{x}_i\right) - \Phi\left(\beta_0 + \boldsymbol{\beta}_2'\boldsymbol{x}_i\right)\right\}.\end{aligned} \tag{4}$$

where $\Phi(\cdot)$ is the standard normal cumulative distribution function. Note that these expressions simplify when only main effects are included in (1) and (2), as $\theta_3 = 0$. Estimation is performed by fitting (1) and (2) to the observed data using standard maximum likelihood estimation and plugging these into (3) and (4). Approximate standard errors for the effects can be obtained using the delta method. The effects in (3) and (4) are marginal, i.e. averaged over the observed covariates in the sample, but can also be calculated conditional on a specific set of covariate values.

2 Outline of the sensitivity analysis method

A brief outline of the sensitivity analysis method is given here, we refer the interested reader to Lindmark et al. [6] for a detailed description. The three types of unobserved confounding for which we perform sensitivity analyses are illustrated in Fig. 1. We will outline the sensitivity analysis for $\mathbf{U}_2$, unobserved mediator-outcome confounding but a similar procedure is used for $\mathbf{U}_1$ and $\mathbf{U}_3$ (see Lindmark et al. [6] for details).

We assume that the error terms in (1) and (2) follow a bivariate standard normal distribution with correlation ρ, the sensitivity parameter. If there is no unobserved mediator-outcome confounding then $\rho = 0$, otherwise $\rho \neq 0$. The sensitivity analysis is performed using a modified maximum likelihood procedure where the joint log-likelihood for M and Y as a function of the regression parameters and ρ is maximized with regards to the regression parameters for a fixed value $\rho = \tilde{\rho}$. The resulting parameter estimates $\hat{\boldsymbol{\beta}}(\tilde{\rho})$ and $\hat{\boldsymbol{\theta}}(\tilde{\rho})$ are then plugged into (3) and (4) to obtain estimates of the natural direct and indirect effects under a given level of unobserved mediator-outcome confounding. This is repeated over a range of ρ. Approximate standard errors for the effects can again be obtained using the delta method.

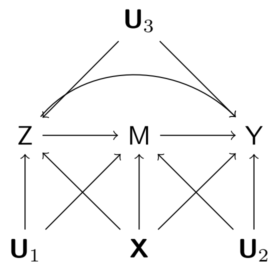

Figure 1: A directed acyclic graph illustrating the three types of unobserved confounding to which sensitivity analysis was performed; $\mathbf{U}_1$, $\mathbf{U}_2$, and $\mathbf{U}_3$.

References

1. Robins JM, Greenland S. Identifiability and Exchangeability for Direct and Indirect Effects. Epidemiology. 1992;3(2):143–155.

2. Pearl J. Direct and Indirect Effects. In: Proceedings of the 17th Conference in Uncertainty in Artificial Intelligence. UAI '01. San Francisco, CA, USA: Morgan Kaufmann Publishers Inc.; 2001. p. 411–420.

3. Imai K, Keele L, Yamamoto T. Identification, inference and sensitivity analysis for causal mediation effects. Stat Sci. 2010;25(1):51–71.

4. VanderWeele TJ, Vansteelandt S. Conceptual issues concerning mediation, interventions and composition. Stat Interface. 2009;2(4):457–468.

5. De Stavola BL, Daniel RM, Ploubidis GB, et al. Mediation analysis with intermediate confounding: Structural equation modeling viewed through the causal inference lens. Am J Epidemiol. 2015;181(1):64–80.

6. Lindmark A, de Luna X, Eriksson M. Sensitivity analysis for unobserved confounding of direct and indirect effects using uncertainty intervals. Stat Med. 2018;37(10):1744–1762.
