# Supplementary material for: Socioeconomic status and survival after stroke – using mediation and sensitivity analyses to assess the effect of stroke severity and unmeasured confounding
Source: BMC Public Health. 2020 Apr 25;20:554. doi: 10.1186/s12889-020-08629-1 (PMC7183587; doi:10.1186/s12889-020-08629-1)
Supplement: Supplementary file 2 — Additional file 2. R code. [file 12889_2020_8629_MOESM2_ESM.pdf]

Additional file 2: R code

Article title: Socioeconomic status and survival after stroke - using mediation and sensitivity analyses to assess the effect of stroke severity and unmeasured confounding
Authors: Anita Lindmark, Bo Norrving, Marie Eriksson

R code for mediation and sensitivity analysis

The mediation and sensitivity analyses were performed using the R package `sensmediation` [1].

```
# Install and load the sensmediation package
install.packages("sensmediation")
library("sensmediation")
```

The probit regression models for the mediator (level of consciousness at hospital arrival) and outcome (death 0-3 months) were estimated using the `glm` function.

```
## Mediator models for ICH and IS: ##
medmod.ICH <- glm(lowered.consc ~ low.income + living.alone + age + I(age^2) + sex + diabetes +
    atrial.fibrillation + smoking, data = data.ICH, family = binomial(link = "probit"))

medmod.IS <- glm(lowered.consc ~ low.income + living.alone + age + I(age^2) + sex + diabetes +
    atrial.fibrillation + smoking, data = data.IS, family = binomial(link = "probit"))

## Outcome models for ICH and IS: ##
outmod.ICH <- glm(death.03m ~ low.income + lowered.consc + living.alone + age + I(age^2) + sex +
    diabetes + atrial.fibrillation + smoking, data = data.ICH, family = binomial(link = "probit"))

outmod.IS <- glm(death.03m ~ low.income + lowered.consc + living.alone + age + I(age^2) + sex +
    diabetes + atrial.fibrillation + smoking, data = data.IS, family = binomial(link = "probit"))
```

Estimation of the natural direct and indirect effects was performed using the `sensmediation()` function with the estimated mediator and outcome models as well as the variable names of the mediator and exposure as input.

```
## Estimation of the NIE and NDE for ICH and IS: ##
effects.ICH <- sensmediation(med.model = medmod.ICH, out.model = outmod.ICH, med.name =
    "lowered.consc", exp.name = "low.income")

effects.IS <- sensmediation(med.model = medmod.IS, out.model = outmod.IS, med.name =
    "lowered.consc", exp.name = "low.income")

# Summary of the results:
summary(effects.ICH)

summary(effects.IS)
```

Sensitivity analysis to the three different kinds of unobserved confounding was performed using the same function with some additional input. A vector of sensitivity parameters was provided through the argument `Rho` and the type of sensitivity analysis performed was regulated using the `type` argument. For sensitivity analysis to unobserved confounding involving the exposure an additional probit model for the exposure given the observed confounders was fit and input. Finally, a printed summary and plots of the results were obtained.

```
## Sensitivity analysis to mediator-outcome confounding: ##
sens.MY.ICH <- sensmediation(med.model = medmod.ICH, out.model = outmod.ICH, med.name =
    "lowered.consc", exp.name = "low.income", type = "my", Rho = seq(-0.9, 0.9, 0.1))

sens.MY.IS <- sensmediation(med.model = medmod.IS, out.model = outmod.IS, med.name =
    "lowered.consc", exp.name = "low.income", type = "my", Rho = seq(-0.9, 0.9, 0.1))

# Summary and plots of the results:
summary(sens.MY.ICH)
plot(sens.MY.ICH)
plot(sens.MY.ICH, effect = "direct")

summary(sens.MY.IS)
plot(sens.MY.IS)
plot(sens.MY.IS, effect = "direct")

## Exposure models: ##
expmod.ICH <- glm(low.income ~ living.alone + age + I(age^2) + sex + diabetes +
    atrial.fibrillation + smoking, data = data.ICH, family =binomial(link = "probit"))

expmod.IS <- glm(low.income ~ living.alone + age + I(age^2) + sex + diabetes + atrial.fibrillation
     + smoking, data = data.IS, family =binomial(link = "probit"))

## Sensitivity analysis to exposure-mediator confounding: ##
sens.ZM.ICH <- sensmediation(med.model = medmod.ICH, out.model = outmod.ICH, med.name =
    "lowered.consc", exp.name = "low.income", exp.model = expmod.ICH, type =  "zm", Rho =
    seq(-0.9, 0.9, 0.1))

sens.ZM.IS <- sensmediation(med.model = medmod.IS, out.model = outmod.IS, med.name =
    "lowered.consc", exp.name = "low.income", exp.model = expmod.IS, type = "zm", Rho =
    seq(-0.9, 0.9, 0.1))

# Summary and plots of the results:
summary(sens.ZM.ICH)
plot(sens.ZM.ICH)
plot(sens.ZM.ICH, effect = "direct")

summary(sens.ZM.IS)
plot(sens.ZM.IS)
plot(sens.ZM.IS, effect = "direct")

## Sensitivity analysis to exposure-outcome confounding: ##
sens.ZY.ICH <- sensmediation(med.model = medmod.ICH, out.model = outmod.ICH, med.name =
    "lowered.consc", exp.name = "low.income", exp.model = expmod.ICH, type = "zy", Rho =
    seq(-0.9, 0.9, 0.1))

sens.ZY.IS <- sensmediation(med.model = medmod.IS, out.model = outmod.IS, med.name =
    "lowered.consc", exp.name = "low.income", exp.model = expmod.IS, type = "zy", Rho =
    seq(-0.9, 0.9, 0.1))

# Summary and plots of the results:
summary(sens.ZY.ICH)
plot(sens.ZY.ICH)
plot(sens.ZY.ICH, effect = "direct")

summary(sens.ZY.IS)
plot(sens.ZY.IS)
plot(sens.ZY.IS, effect = "direct")
```

References

1. Lindmark A. sensmediation: Parametric Estimation and Sensitivity Analysis of Direct and Indirect Effects; 2018. R package version 0.3.0. Available from: http://cran.R-project.org/package=sensmediation.
