# Supplementary material for: Socioeconomic status and survival after stroke – using mediation and sensitivity analyses to assess the effect of stroke severity and unmeasured confounding
Source: BMC Public Health. 2020 Apr 25;20:554. doi: 10.1186/s12889-020-08629-1 (PMC7183587; doi:10.1186/s12889-020-08629-1)
Supplement: Supplementary file 3 — Additional file 3. Tables. [file 12889_2020_8629_MOESM3_ESM.pdf]

Additional file 3: Tables

Article title: Socioeconomic status and survival after stroke - using mediation and sensitivity analyses to assess the effect of stroke severity and unmeasured confounding
Authors: Anita Lindmark, Bo Norrving, Marie Eriksson

1 Probit regression models used in main analyses

Table 1: Probit models for the mediator lowered consciousness at hospital arrival and outcome death 0-3 months, by stroke type. Estimated regression parameters with 95% CIs in parentheses.

	ICH		IS	
	Lowered consciousness	Death 0-3 months	Lowered consciousness	Death 0-3 months
Intercept	0.261	-1.956	0.117	-1.677
	$(-0.243, 0.765)$	$(-2.646, -1.267)$	$(-0.202, 0.437)$	$(-2.159, -1.195)$
Low income	0.115	0.055	0.065	0.041
	$(0.048, 0.182)$	$(-0.023, 0.133)$	$(0.033, 0.097)$	$(0.006, 0.077)$
Lowered consciousness		1.363		1.208
		$(1.289, 1.436)$		$(1.168, 1.247)$
Male	-0.148	0.037	-0.071	0.042
	$(-0.214, -0.082)$	$(-0.041, 0.114)$	$(-0.103, -0.039)$	$(0.005, 0.078)$
Age	-0.023	-0.012	-0.058	-0.038
	$(-0.039, -0.008)$	$(-0.033, 0.008)$	$(-0.067, -0.049)$	$(-0.051, -0.025)$
Age^2	< 0.001	< 0.001	< 0.001	< 0.001
	(<0.001, <0.001)	(<0.001, <0.001)	(<0.001, 0.001)	(<0.001, 0.001)
Atrial fibrillation	0.052	0.279	0.352	0.223
	$(-0.031, 0.135)$	$(0.187, 0.371)$	$(0.319, 0.384)$	$(0.188, 0.259)$
Diabetes	0.052	0.095	0.054	0.149
	$(-0.038, 0.142)$	$(-0.009, 0.198)$	$(0.016, 0.092)$	$(0.108, 0.190)$
Smoking status				
Unknown	0.636	0.561	0.404	0.413
	$(0.540, 0.731)$	$(0.455, 0.667)$	$(0.351, 0.457)$	$(0.356, 0.469)$
Smoker	0.027	0.111	0.021	0.051
	$(-0.077, 0.131)$	$(-0.015, 0.237)$	$(-0.026, 0.068)$	$(-0.005, 0.107)$
Living alone	0.002	-0.056	0.088	0.050
	$(-0.063, 0.068)$	$(-0.134, 0.021)$	$(0.056, 0.121)$	$(0.014, 0.086)$

Table 2: Probit models for the exposure low income, by stroke type. Estimated regression parameters with 95% CIs in parentheses.

	**ICH**	**IS**
Intercept	1.296	1.111
	$(0.790, 1.802)$	$(0.856, 1.365)$
Male	-0.632	-0.665
	$(-0.698, -0.567)$	$(-0.689, -0.640)$
Age	-0.048	-0.046
	$(-0.064, -0.033)$	$(-0.054, -0.039)$
Age^2	< 0.001	< 0.001
	(<0.001, 0.001)	(<0.001, <0.001)
Atrial fibrillation	-0.051	-0.002
	$(-0.136, 0.034)$	$(-0.029, 0.025)$
Diabetes	0.093	0.101
	$(0.001, 0.185)$	$(0.072, 0.131)$
Smoking status		
Unknown	0.071	0.082
	$(-0.028, 0.170)$	$(0.034, 0.129)$
Smoker	0.048	0.108
	$(-0.058, 0.153)$	$(0.074, 0.143)$
Living alone	-0.078	-0.139
	$(-0.146, -0.011)$	$(-0.164, -0.113)$

2 Models and results with adjustment for level of education

Table 3: Probit models for the mediator (lowered consciousness at hospital arrival) and the outcome (death 0-3 months after stroke) with adjustment for education level for patients with intracerebral hemorrhage (ICH) and ischemic stroke (IS). Estimated regression parameters with 95% CIs in parentheses.

	**ICH**		**IS**	
	Lowered consciousness	Death 0-3 months	Lowered consciousness	Death 0-3 months
Intercept	0.255	−1.761	0.168	−1.640
	(-0.263, 0.773)	(-2.476, -1.057)	(−0.159, 0.496)	(−2.136, −1.144)
Low income	0.110	0.021	0.052	0.032
	(0.042, 0.179)	(-0.060, 0.102)	(0.019, 0.086)	(−0.005, 0.068)
Lowered consciousness		1.368		1.207
		(1.294, 1.442)		(1.167, 1.247)
Male	−0.150	0.028	−0.070	0.040
	(-0.217, -0.084)	(-0.051, 0.107)	(−0.103, −0.037)	(0.003, 0.077)
Age	−0.023	−0.015	−0.058	−0.037
	(-0.039, -0.007)	(-0.036, 0.006)	(−0.068, −0.049)	(−0.051, −0.024)
Age^2	< 0.001	< 0.001	< 0.001	< 0.001
	(<0.001, <0.001)	(<0.001, <0.001)	(<0.001, 0.001)	(<0.001, 0.001)
Atrial fibrillation	0.046	0.257	0.352	0.222
	(-0.038, 0.130)	(0.164, 0.351)	(0.319, 0.385)	(0.186, 0.258)
Diabetes	0.043	0.094	0.052	0.147
	(-0.048, 0.134)	(-0.011, 0.198)	(0.013, 0.090)	(0.105, 0.188)
Smoking status				
Unknown	0.635	0.560	0.396	0.409
	(0.539, 0.731)	(0.453, 0.667)	(0.343, 0.450)	(0.352, 0.466)
Smoker	0.027	0.096	0.016	0.050
	(-0.077, 0.132)	(-0.031, 0.224)	(−0.032, 0.063)	(−0.007, 0.107)
Living alone	−0.007	−0.069	0.085	0.046
	(-0.073, 0.060)	(-0.147, 0.009)	(0.052, 0.117)	(0.010, 0.083)
Education level				
Secondary school	−0.001	−0.062	−0.035	−0.060
	(-0.073, 0.071)	(-0.145, 0.022)	(−0.070, −0.001)	(−0.097, −0.022)
University	0.001	−0.197	−0.078	−0.109
	(-0.088, 0.091)	(-0.305, -0.089)	(−0.124, −0.031)	(−0.162, −0.056)

Table 4: Estimated total ($\widehat{\text{TE}}$), natural indirect ($\widehat{\text{NIE}}$) and direct effects ($\widehat{\text{NDE}}$) on the absolute risk difference scale (%) and proportion mediated (%), with adjustment for education level. 95% CIs in parentheses.

	$\widehat{\text{TE}}$	$\widehat{\text{NIE}}$	$\widehat{\text{NDE}}$	Proportion mediated^a
**ICH**	2.2	1.7	0.5	77.0
	(−0.1, 4.5)	(0.6, 2.8)	(−1.5, 2.5)	
**IS**	0.7	0.3	0.5	39.5
	(0.2, 1.3)	(0.1, 0.5)	(−0.1, 1.0)	

$^a 100 \times \widehat{\text{NIE}}/\widehat{\text{TE}}$
